# Foreskin-derived mesenchymal stromal cells with aldehyde dehydrogenase activity: isolation and gene profiling

**DOI:** 10.1186/s12860-018-0157-0

**Published:** 2018-04-06

**Authors:** Mehdi Najar, Emerence Crompot, Leo A. van Grunsven, Laurent Dollé, Laurence Lagneaux

**Affiliations:** 1 Laboratory of Clinical Cell Therapy, Jules Bordet Institute, Université Libre de Bruxelles (ULB), Campus Erasme, Bâtiment de Transfusion (Level +1), Route de Lennik 808, 1070 Brussels, Belgium; 2 Liver Cell Biology Laboratory, Vrije Universiteit Brussel, Brussels, Belgium

**Keywords:** Foreskin mesenchymal stromal cells, Aldehyde dehydrogenase activity, Fluorescence activated cell sorting, Transcriptome analysis

## Abstract

**Background:**

Mesenchymal stromal cells (MSCs) become an attractive research topic because of their crucial roles in tissue repair and regenerative medicine. Foreskin is considered as a valuable tissue source containing immunotherapeutic MSCs (FSK-MSCs).

**Results:**

In this work, we used aldehyde dehydrogenase activity (ALDH) assay (ALDEFLUOR™) to isolate and therefore characterize subsets of FSK-MSCs. According to their ALDH activity, we were able to distinguish and sort by fluorescence activated cell sorting (FACS) two subsets of FSK-MSCs (referred as ALDH^+^ and ALDH^−^). Consequently, these subsets were characterized by profiling the gene expression related to the main properties of MSCs (proliferation, response to hypoxia, angiogenesis, phenotype, stemness, multilineage, hematopoiesis and immunomodulation). We thus demonstrated by Real Time PCR several relevant differences in gene expression based on their ALDH activity.

**Conclusion:**

Taken together, this preliminary study suggests that distinct subsets of FSK-MSCs with differential gene expression profiles depending of ALDH activity could be identified. These populations could differ in terms of biological functionalities involving the selection by ALDH activity as useful tool for potent therapeutic applications. However, functional studies should be conducted to confirm their therapeutic relevance.

**Electronic supplementary material:**

The online version of this article (10.1186/s12860-018-0157-0) contains supplementary material, which is available to authorized users.

## Background

Mesenchymal stromal cells are attractive for regenerative medicine and immunotherapy due to their multilineage and immunomodulatory potential [1–3]. However, the application of MSCs in cell therapy is often limited by low cell numbers and low proliferation rates. Therefore, finding a suitable cell source has been a major challenge in recent years [4]. Although MCSs are primarily isolated from bone marrow (BM) [5], several tissues, such as adipose tissue (AT) [6], cord blood (CB) [7], dental pulp [8] and placenta [9], have been proposed as alternative sources. Skin has also been reported to be a source of MSCs [10], and foreskin (FSK) seems to be particularly enriched in MSCs [11]. Therefore, we previously demonstrated that FSK-MSCs are a more suitable cellular product for cell therapy [12]. Though previously regarded as waste, these cells have shown great potential for stem cell-based anti-tumor therapy [13, 14]. In this regard, it is important to establish distinct subsets of MSCs with specific therapeutic features to be used for cell-targeted therapy. Although many studies have addressed the isolation and characterization of sub-populations of MSCs [6, 15, 16], little is known about FSK-MSCs. Aldehyde dehydrogenase (ALDH) activity is increasingly being used as a means to isolate stem or progenitor cells [17, 18]. ALDH enzymes are involved in several cellular properties such as self-protection, proliferation and differentiation [17, 19–23].

In the present study, we combined the ALDEFLUOR™® assay [24] and fluorescence activated cell sorting (FACS) [25] to identify and isolate MSCs based on their ALDH activity. Consequently, two populations of FSK-MSCs (referred to as ALDH^+^ and ALDH^−^) were obtained and characterized based on their gene expression profiles in relation to the major properties of MSCs. Thus, we highlight differential gene expression profiles with respect to stemness, proliferation, phenotype, immunomodulation, hypoxia response ability, hematopoietic support and multilineage capacity according to ALDH activity in FSK-MSC subsets.

In summary, 2 distinct subsets of FSK-MSCs that harbor specific gene profiles can be identified based on their ALDH activity. Conducting functional studies may therefore confirm therapeutic relevance.

## Results

### ISCT compliance of FSK-MSCs

**Fig. 1:**
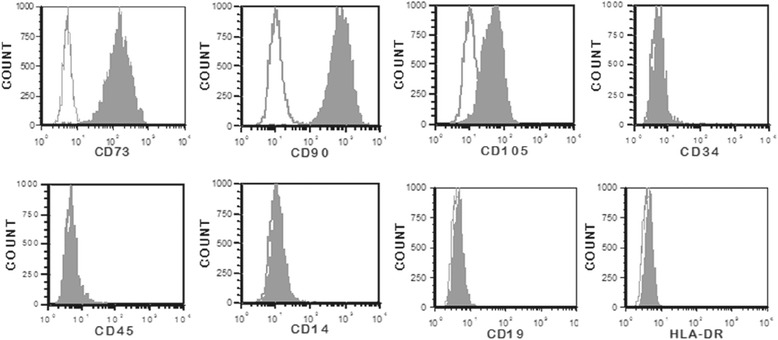
Representative flow cytometry analysis of FSK-MSC immunophenotype. We used a panel of fluorochrome labelled monoclonal antibodies (mAbs). Empty histograms show the background staining with isotype control mAbs, and solid histograms represent specific staining of the indicated cell surface markers

**Fig. 2:**
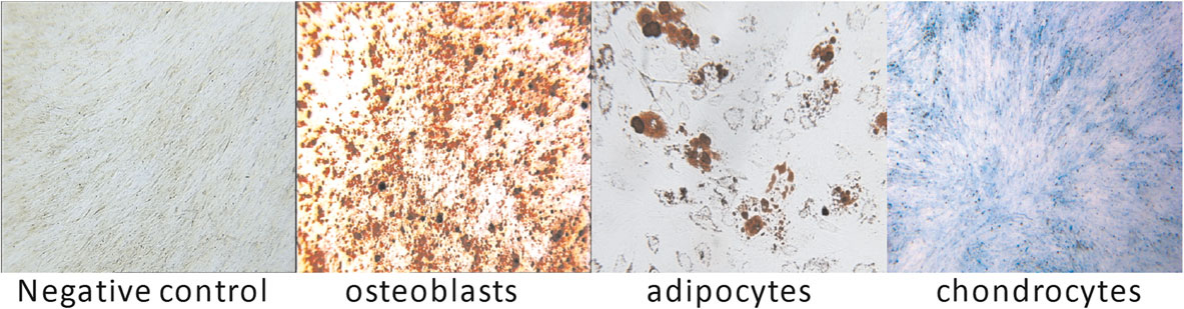
Representative images illustrating the MSC multilineage potential. Each lineage differentiation was assessed by using both specific induction medium and staining techniques

The cells from FSK cultures showed the typical fibroblast-like shape with a high capacity to adhere to plastic. Immunophenotyping of the cells by flow cytometry demonstrated positivity (> 95%) for CD73, CD90 and CD105 but lack (< 5%) of CD14, CD19, CD34, CD45 and HLA-DR expression (Fig. 1). Moreover, these cells exhibited a multilineage potential in-vitro. After 21 days of osteogenic induction, calcium deposits were observed by Alizarin red staining. After 10 days of adipogenic induction, lipid vacuoles were revealed by Oil Red O staining. After 21 days of chondrogenic induction, proteoglycans were shown by Alcian blue staining (Fig. 2). Accordingly, FSK-MSCs used in this study present the basic characteristics of MSCs.

### Sorting and isolation of FSK-MSCs based on ALDH activity

Two populations with high and low ALDH enzymatic activity were isolated (ALDH^+^ and ALDH^−^). ALDH activity was significantly different in these 2 populations: 0.45 ± 0.05 UA (Arbitrary unity) for the ALDH^−^ and 21.78 ± 1.01 UA for the ALDH^+^ population, *p* = 0.0020 (Additional file 1: Figure S1).

### Gene expression analysis of ALDH-sorted FSK-MSC subsets

#### Stemness (Nanog; octamer-binding transcription factor 4(Oct4); Rex1) (Fig. 3)

Sorting for ALDH activity allowed us to distinguish ALDH^+^ and ALDH^−^ subsets based on the expression of genes related to stemness. We did not observe *Nanog* expression in either subset. We observed the up-regulation of *Oct4* (235.5 ± 17.4) in ALDH^+^ cells compared with ALDH^−^ (173.2 ± 5) cells (*p* = 0.0362). *Rex1* expression was similar between both subsets, with a slight but not significant decrease in the ALDH^−^ population (3259 ± 147.5 in ALDH^+^ vs 2759 ± 56.9 for ALDH^−^).

**Fig. 3:**
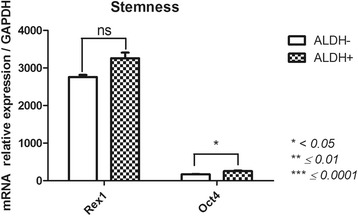
Stemness-related gene expression profile of FSK-MSC subsets according to ALDH activity. After flow cytometry sorting of ALDH^+^ and ALDH^−^ FSK-MSC subsets, we investigated by qPCR the expression profiles of pluripotency-associated genes Oct4, Nanog and Rex1. Data are presented as mean ± SEM of mRNA gene expression relative to *GAPDH* expression. *(ns = non significant)*

#### Proliferation/cell cycle (CyclinA (CCNA), CCNB, CCNE; CDK1, CDK2, Fos proto-oncogene (FosB); p21; p53; p16; retinoblastoma protein (pRB); cell division cycle 25A (CDC25A); signal transducer and activator of transcription (STAT1)) (Fig. 4)

ALDH^+^ and ALDH^−^ subsets demonstrated distinct gene profiles associated with proliferation/cell cycle. The ALDH^+^ subset displayed significantly higher expression of these genes. The most highly expressed genes in ALDH^+^ cells vs ALDH^−^ cells were p21 (71,794 ± 811.2 vs 50,446 ± 466.7; *p* = 0.0033), CDK1 (22,788 ± 605.1 vs 19,097 ± 415.7; *p* = 0.0219) and CCNA (29,338 ± 415.1 vs 12,854 ± 645.1; *p* = 0.0041). We observed significantly higher expression of CDK2, pRB and CDC25A in ALDH^+^ cells (6683 ± 141.5, 7643 ± 202.1, 1140 ± 24.9) cells than in ALDH^−^ cells (4722 ± 50.6, 5388 ± 113, 505.2 ± 47.1) (*p* = 0.0055, p = 0.0033, *p* = 0.0014). We also noticed weakly increased of *FosB* gene expression in ALDH^+^ cells (2592 ± 30.4) compared with ALDH^−^ cells (2283 ± 96.3). The ALDH^+^ population expressed significantly higher levels of *CCNB* and *CCNE* than the ALDH^−^ population (1532 ± 80.5 vs 1169 ± 43.9, 773.5 ± 92.7 vs 351.8 ± 14.5 with *p* = 0.0112 and *p* = 0.0364, respectively). Moreover, *STAT1* was more highly expressed in the ALDH^+^ population (1286 ± 41.9 vs 858.2 ± 58.1, *p* = 0.0337).

**Fig. 4:**
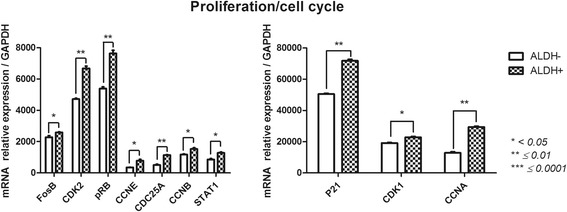
Cell-cycle-related gene expression profile of FSK-MSC subsets according to ALDH activity. After flow cytometry sorting of ALDH+ and ALDH- FSK-MSC subsets, we investigated by qPCR the expression profiles of cell-cycle-associated genes *FosB, CDK2, pRB, CCNE, CDC25A, CCNA, CCNB, STAT1, P21 and CDK1*. Data are presented as mean ± SEM of mRNA gene expression relative to *GAPDH* expression

#### Phenotype (melanoma cell adhesion molecule CD146 (MCAM); CD200; vascular cell adhesion molecule 1 CD106 (VCAM-1); intercellular adhesion molecule 1 CD54 (ICAM-1); CD58 (LFA-3)) (Fig. 5)

ALDH^+^ and ALDH^−^ subsets showed several gene expression differences with respect to the MSC phenotype. First, CD146 and CD200 were not found in either subset. CD54, CD58 and CD106 were most highly expressed in ALDH^+^ cells (5218 ± 12.1, 9135 ± 52.7, 550.4 ± 16.8 respectively) compared with ALDH^−^ cells (4403 ± 12.9, 7211 ± 30.2, 148.3 ± 10.6, respectively) with significant *p*-values (*p* = 0.0008, *p* = 0.0013, *p* = 0.0003, respectively).

**Fig. 5:**
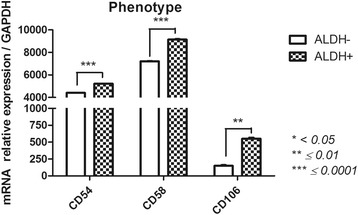
Phenotype-related gene expression profile of FSK-MSC subsets according to ALDH activity. After flow cytometry sorting of ALDH+ and ALDH- FSK-MSC subsets, we investigated by qPCR the expression profiles of phenotype-associated genes *CD54, CD58 and CD106*. Data are presented as mean ± SEM of mRNA gene expression relative to *GAPDH* expression

#### Hypoxia response (hypoxia-inducible factor (HIF)-1α; HIF-2α; solute carrier family 2 member 1 (GLUT1)) (Fig. 6)

Consistent with their ALDH activity, the expression of genes involved in the hypoxia response was significantly different between the ALDH^+^ and ALDH^−^ subsets. *HIF1α, HIF2α* and *GLUT1* expression levels were higher in ALDH^+^ cells (675.5 ± 36.6 vs 529.7 ± 8.1, 17,258 ± 431.9 vs 7354 ± 341.3, 53,748 ± 3251 vs 37,335 ± 3397, respectively), and all had significant p-values except for *HIF1α* (*p* = 0.0525, *p* = 0.0027, *p* = 0.0057, respectively).

**Fig. 6:**
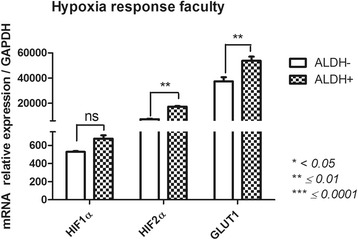
Hypoxia-related gene expression profile of FSK-MSC subsets according to ALDH activity. After flow cytometry sorting of ALDH+ and ALDH- FSK-MSC subsets, we investigated by qPCR the expression profiles of hypoxia-associated genes *HIF1α, HIF2α and GLUT1*. Data are presented as mean ± SEM of mRNA gene expression relative to *GAPDH* expression. *(ns = non significant)*

#### Angiogenesis (angiopoietin (ANG)1, ANG2, Fms-related tyrosine kinase 1 (FLT1); vascular endothelial growth factor (VEGF)) (Fig. 7)

ALDH^+^ and ALDH^−^ cells had distinct gene expression profiles associated with angiogenesis. ANG2 was not expressed by either subset. Compared with ALDH^−^ cells, ALDH^+^ cells showed significantly higher levels of ANG1 (887.3 ± 9.2 vs 498.5 ± 12.9), FLT1 (57.87 ± 17.8 vs 2867 ± 14.5) and VEGF (18,854 ± 508.6 vs 15,098 ± 429.2). Thus, ALDH^+^ cells appear to exhibit highly angiogenic properties.

**Fig. 7:**
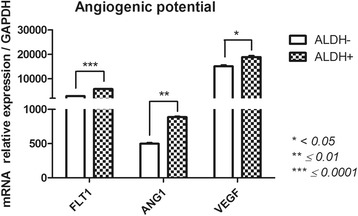
Angiogenesis-related gene expression profile of FSK-MSC subsets according to ALDH activity. After flow cytometry sorting of ALDH+ and ALDH- FSK-MSC subsets, we investigated by qPCR the expression profiles of angiogenesis-associated genes *FLT1, ANG1 and VEGF*. Data are presented as mean ± SEM of mRNA gene expression relative to *GAPDH* expression

#### Hematopoietic support (matrix metalloproteinase 2 (MMP2); stromal derived factor 1 (SDF1); kit ligand (SCF); Interleukin-6 (IL-6); IL-8) (Fig. 8)

ALDH^+^ and ALDH^−^ cells subsets showed significant differences in genes linked to the hematopoietic supporting capacity of FSK-MSCs. *MMP2, SCF, SDF1, IL-6 and IL-8* were strongly expressed in the ALDH^+^ subset (762,594 ± 68,274, 62,691 ± 4273, 155,209 ± 6358, 142,246 ± 1405 and 41,120 ± 806.3) compared with ALDH^−^ cells (404,009 ± 6630, 30,176 ± 800.4, 130,426 ± 2144, 78,498 ± 2771 and 33,115 ± 1102) (*p* = 0.0393, *p* = 0.0124, *p* = 0.0324, *p* = 0.0026 and *p* = 0.0018, respectively).

**Fig. 8:**
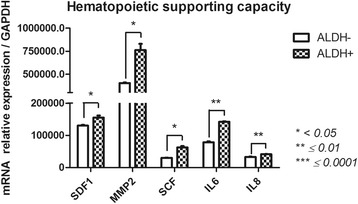
Pro-hematopoietic-related gene expression profile of FSK-MSC subsets according to ALDH activity. After flow cytometry sorting of ALDH+ and ALDH- FSK-MSC subsets, we investigated by qPCR the expression profiles of pro-hematopoietic-associated genes *SDF1, MMP2, SCF, IL6 and IL8*. Data are presented as mean ± SEM of mRNA gene expression relative to *GAPDH* expression

#### Immunomodulation capacity (Galectin1 (GAL1); hepatocyte growth factor (HGF); leukemia inhibitory factor (LIF); cyclooxygenase (COX)1 and COX2) (Fig. 9)

ALDH^+^ and ALDH^−^ subsets had different immunoregulatory gene expression patterns. These genes were more highly expressed in ALDH^+^ cells than in ALDH^−^ subsets: 1.1 × 10^6^ ± 23,780 vs 526,797 ± 39,702 for GAL1 (*p* = 0.0077), 35,740 ± 2110 vs 17,626 ± 1027 for *COX1* (*p* = 0.0056), 20,484 ± 366.2 vs 16,219 ± 551.6 for *COX2* (*p* = 0.0282), 2655 ± 84 vs 850.7 ± 69.2 for *HGF* (*p* = 0.0067) and 2615 ± 161.8 vs 1236 ± 129.8 for LIF (*p* = 0.0133).

**Fig. 9:**
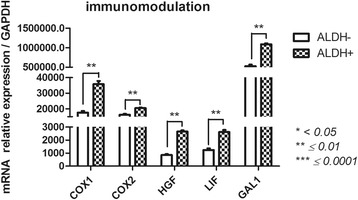
Immunomodulatory-related gene expression profile of FSK-MSC subsets according to ALDH activity. After flow cytometry sorting of ALDH+ and ALDH- FSK-MSC subsets, we investigated by qPCR the expression profiles of pro-hematopoietic-associated genes *COX1, COX2, HGF, LIF and GAL1*. Data are presented as mean ± SEM of mRNA gene expression relative to *GAPDH* expression

#### Multilineage competence (Fig. 10)

A. Chondrogenesis potential (Sry box 9 (Sox9); Collagen type II alpha 1 chain (Col2a1); Aggrecan (ACAN); Cartilage Oligomeric matrix protein (COMP)).

**Fig. 10:**
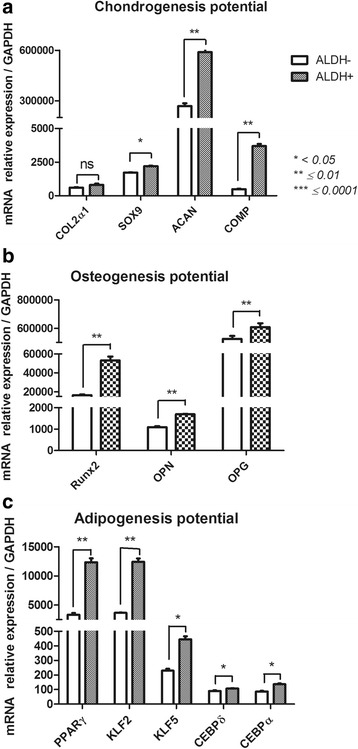
Multilineage-related gene expression profile of FSK-MSC subsets according to ALDH activity. After flow cytometry sorting of ALDH+ and ALDH- FSK-MSC subsets, we investigated by qPCR the expression profiles of multilineage-associated genes such as COL2a1, SOX9, CAN, COMP for chondrogenesis (**a**), Runx2, OPN, OPG for osteogenesis (**b**) and PPARγ, KLF2, KLF5, CEBPα, CEBPδ for adipogenesis (**c**). Data are presented as mean ± SEM of mRNA gene expression relative to *GAPDH* expression. *(ns = non significant)*

ALDH^+^ and ALDH^−^ cells demonstrated distinct gene expression profiles with respect to chondrogenic potential. We observed no significant difference between these subsets regarding *COL2α1* expression. Meanwhile, *ACAN* (589,668 ± 21,737 vs 268,260 ± 16,493, *p* = 0.0059), Sox9 (2206 ± 23.8 vs 1715 ± 30.5, *p* = 0.0114 and COMP (3702 ± 156.4 vs 487.5 ± 30.3, *p* = 0.0021) expression were significantly higher in ALDH^+^ cells than in ALDH^−^ cells.

B. Osteogenesis potential (Runt related transcription factor 2 (Runx2); Osterix (OSX); Integrin binding sialoprotein (BSP); TNF receptor superfamily member 11 (OPG); Secreted phosphoprotein type 1 (OPN)).

The osteogenic gene profiles of ALDH^+^ and ALDH^−^ cell subsets were substantially different. BSP and OSX were not expressed in either subset. Runx2 (53,180 ± 3959 vs 16,386 ± 619.6, *p* = 0.0089), OPG (609,334 ± 25,983 vs 526,399 ± 20,205, *p* = 0.0064) and OPN (1698 ± 14 vs 1093 ± 44.8, *p* = 0.0026) were more highly expressed in ALDH^+^ cells than in ALDH^−^ cells.

C. Adipogenesis potential (CCAAT/enhancer binding protein alpha (C/EBP-α), C/EBP-δ; Peroxisome proliferator activated receptor gamma (PPAR-γ); Kruppel like factor (KLF2); KLF5; Adiponectin (ADIPOQ)).

The majority of the genes linked to adipogenesis were differentially expressed by the ALDH^+^ and ALDH^−^ subsets. *ADIPOQ* was not expressed at all. Adipogenesis genes were most highly expressed in ALDH^+^ cells: 12323 ± 684.3 vs 3332 ± 306 for PPARγ, 12,411 ± 564.1 vs 3666 ± 43.9 for KLF2, 445.9 ± 20.2 vs 229.8 ± 12.9 for KLF5, 136.1 ± 6.1 vs 86.9 ± 4.5 for CEBPα and 106.6 ± 3.2 vs 90.9 ± 4 for CEBPδ (*p* = 0.0019, *p* = 0.0043, *p* = 0.0221, *p* = 0.0194 and *p* = 0.0260).

## Discussion

MSCs can be isolated from both adult and fetal tissues [26–29] and may share basic characteristics [30]. However, these MSCs may have some differences probably related to their distinct gene and protein profiles [31, 32]. As a potential alternative source of MSCs, FSK tissue has recently emerged as a promising candidate for different cell-based therapies [12, 13, 33]. Consequently, we have demonstrated that FSK contains a high number of immunotherapeutic MSCs [12]. However, different sub-populations of MSCs with specific features might be observed [6]. Of Importance, understanding the product profile of the intended therapy is crucial to achieving the desired therapeutic effect [34, 35]. Different enrichment methods to obtain MSCs are reported in the literature with contrasting results [36]. Thus, no single surface marker is currently available to identify and isolate MSCs from various tissue environments that could be therapeutically relevant [37]. Therefore, finding a suitable method to identify subsets of MSCs with distinct features is of high priority to ensure efficient therapy.

In this regard, ALDH activity has been shown to be important for the biology of stem and progenitor cells [17]. In MSCs, ALDH activity is critical for tissue repair and regeneration [38–40]. In addition, ALDH activity and expression appear to be promising markers and potential therapeutic targets for treating many malignancies [41]. A recent study reported that low expression of ALDH in decidual MSC (DMSC) isolated from preeclamptic (PE) patients is associated with reduced resistance to cell toxicity. In line, stem and progenitor cells expressing high level of ALDH were shown to be clinically safe and effective cell therapy for peripheral ischemia [42]. These cyto-protective enzymes may thus potentiate cell therapy by serving as markers of highly therapeutic MSCs that harbor specific competencies.

Using FACS, we were able to sort two subsets of FSK-MSCs (e.g., ALDH^+^ and ALDH^−^) based on their ALDH activity. Several tissue-dependent progenitors with high ALDH activity have been reported to be therapeutically relevant because they harbor specific biological functions such as tissue repair and hematopoietic reconstitution [19]. The global gene expression profiles of a variety of non-hematopoietic progenitors are likely to have an important impact on their properties as well as on their cellular therapeutic applications [43]. Accordingly, depending on their ALDH activity, these FSK-MSC subsets demonstrated distinct transcriptional profiles for genes associated with major MSC properties (e.g., proliferation, response to hypoxia, angiogenesis, phenotype, stemness, multilineage, hematopoiesis, and immunomodulation) [8, 44–47]. As discussed below, these gene expression differences between FSK-MSCs subsets may indicate specific biological features with considerable therapeutic relevance.

In terms of stemness [48], Nanog was not expressed in either FSK-MSC subset, while Rex1 and Oct4 were more highly expressed in ALDH^+^ cells than in ALDH^−^ cells. These genes appear to be crucial for the efficient maintenance of cell pluripotent identity [49]. Our observations were consistent with the work of Dey and colleagues, which suggested that fetal heart-derived cells with high ALDH activity exhibit enhanced gene expression for self-renewal, proliferation, and survival [50].

Regarding the cell cycle, we observed substantial differences in FosB, CDK1, CDK2, pRB, CCNA, CCNB, CCNE, CDC25A and p21 expression. All of these targets were highly upregulated in ALDH^+^ cells. The cell cycle is likely to be influenced by ALDH activity and is likely critical for cell specificity and the properties of different cellular populations [45, 51, 52]. Mechanistically, ALDH has been implicated in controlling cell proliferation during the catabolism of endogenous substrates that have the capacity to either stimulate or inhibit the expression of genes involved in the cell cycle [53].

An important issue for optimal cell-based therapy is the phenotype of the cellular product being used [54]. Progenitors cells generally express similar markers regardless of their tissue of origin, with difference being largely restricted to CD54, 58, 106, 146 and CD200 [18, 55–57]. Here, we notably observed increased CD54, 58 and 106 expressions in ALDH^+^ FSK-MSCs. The expression of the primary adhesion molecules CD54 and CD58 has been reported to vary according to the MSC source [18, 19]. Cells with CD54 expression were shown to have high immunosuppressive capacity [58]. Interestingly, CD106 expression was even restricted to specific tissues [59] and was suggested to have distinct immunomodulatory abilities [60] and pro-angiogenic potential [61]. The absence of CD146 and CD200 expression in FSK-MSCs subsets is likely dependent on the tissue of origin and the MSC environment [56, 62, 63]. The enrichment of CD200 may also have a significant impact on immunomodulation [55], regenerative potential [64] and osteogenesis in a subset of progenitor cells [63]. As a mesenchymal marker, CD146 was shown to define subsets of cells of different origin with remarkable stemness, multilineage potential and immunomodulatory capacities [65].

During tissue repair, the hypoxia-induced activation of HIFs regulates different cellular functions such as angiogenesis and inflammation [66, 67]. In ALDH^+^ cells, a significant increase in HIF2α, GLUT1 and, to a lesser extent, HIF1α expression was observed. Gene responses to hypoxia are cell-type specific, and HIFs have been implicated in stemness [68, 69]. In parallel, high ALDH activity enhanced stem-cell features in breast cancer cells by activating HIF2α expression [70], suggesting a crucial role for ALDH in targeted therapies. With regard to hypoxia, ALDH expression in MSCs is required for cellular resistance to oxidative stress [38]. ALDH activity clearly influences HIF gene expression patterns to allow for appropriate MSC responses to hypoxia. The expression of HIF1α and GLUT1 genes was higher in ALDH^+^ cells confirming their importance for cell-response to hypoxia. Thus, the therapeutic efficiency of ALDH^+^ cells was observed in patients with chronic myocardial ischemia and critical limb ischemia [71, 72].

Based on their ALDH activity, MSCs promote angiogenesis as a part of their tissue repair and regeneration capability [66]. MSCs with high ALDH activity are likely to be more responsive to hypoxia by upregulating FLT1, CXCR4, and ANG2 [56]. Indeed, we observed increased expression of angiogenic genes (ANG1, FLT1 and VEGF) in ALDH^+^ cells. However, ANG2 was not expressed in FSK-MSCs. Vishnubalaji and colleagues demonstrated that FSK-MSCs exhibit high tube-forming capability (similar to endothelial cells from the human umbilical vein) [56]. MSCs from non-FSK tissues that harbor high ALDH activity were shown to have the ability to home to damaged tissues and promote angiogenesis, thus protecting mice against acute ischemic injury [19]. By showing significantly higher levels of ANG1, FLT1 and VEGF, ALDH^+^ cells may exhibit potent angiogenic properties that have proved to be therapeutically relevant. Indeed, human BM ALDH^+^ cells were able to improve perfusion in ischemic limbs after transplantation (showed augmented recovery of perfusion and increased blood vessel density) [73].

MSCs also play an important role in the homeostasis of the hematopoietic system by generating most of the stromal cells that are present in the hematopoietic stem cell (HSC) niche and by providing different factors that regulate hematopoiesis, such as IL6, IL8, MMP2 and SCF [74]. We found that ALDH^+^ FSK-MSC subsets may exhibit potent hematopoiesis-supporting abilities following IL6, IL8, SCF, SDF1 and MMP2 up-regulation and these results corroborate the fact that ALDH activity may be used as a surrogate marker for hematopoietic stem cell transplant activity [75]. As suggested previously, IL-8, rather than IL-6, may mechanistically support the engraftment of repopulating cells by enhancing MMP-2 expression and therefore increasing migration and infiltration within BM [76].

FSK-MSCs are likely to be considered immunotherapeutic cells [12], and their immunomodulatory properties may actively account for their tissue repair activity [77]. MSCs can modulate the immunological functions of several immune cell populations both in vitro and in vivo [78, 79]. However, distinct types of MSCs have been shown to display specific immunomodulatory characteristics [9, 80]. Here, ALDH^+^ FSK-MSCs subsets showed elevated COX1/2, GAL1, LIF and HGF expression, suggesting better immunomodulatory features that might be more attractive for immunotherapeutic interventions. Indeed, all of these factors were previously described to actively compete in establishing a tolerogenic state sustained by MSCs [81]. Accordingly, ALDH was shown to be critical for the induction of Tregs, thus promoting immunological tolerance [82].

FSK-MSCs are capable of differentiating into several lineages and are thus relevant for regenerative cell therapy [14, 57]. We have therefore examined the expression of the major genes associated with the tri-lineage potential of MSCs [83, 84]. For osteogenesis, we did not observe OSX or BSP expression in either subset, whereas OPG, OPN and Runx2 were more highly expressed in ALDH^+^ cells, suggesting that this population has a higher capacity to differentiate into osteoclasts than ALDH^−^ cells. Moreover, FSK cells have been shown to display osteogenesis capacity and participate to bone repair [85, 86]. De Kock and colleagues demonstrated that FSK cells are the most appropriate stem cells for bone-based applications compared with other types of MSCs [43]. Increased ALDH levels are associated with enhanced stress resistance and muscle regeneration capacity in muscle-derived cell progenitors [84]. ALDH^+^ MSCs from umbilical cord blood had a greater ability to differentiate and their transplantation into fractured mouse femurs enabled early repair of tissues and rapid bone substitution [87]. Compared to cells with low activity (ALDH^−^), the ALDH^+^ sub-population of murine [88] and canine [89] cells exhibited a higher capacity for osteogenic and adipogenic differentiation.

As for adipogenesis, Li and colleagues showed that FSK-MSCs can be differentiated into adipocytes and osteocytes [13]. This ability appeared to be more prominent in ALDH^+^ FSK-MSCs, which expressed significantly higher levels of adipogenic markers such as CEBPα, CEBPδ, PPARγ, KLF2, and KLF5 [90–92].

Finally, we evaluated the ability of FSK-MSCs to differentiate into chondroblasts [93, 94]. ALDH^+^ cells expressed higher levels of chondrogenic genes (Sox9, ACAM, COMP). However, ALDH activity may not be relevant for chondrogenesis capacity in MSCs [95]. These observations suggest that ALDH^+^ FSK-MSC subsets may differ in their differentiation capacity depending on the tissue context and may thus play a significant role in regenerative medicine. As discussed by Dollé et al. [96], although ALDH isoforms may be used as cell markers, they play different roles in stem/progenitor cell populations.

## Conclusion

In the present work, the use of ALDH activity to identify FSK-MSC subsets with specific gene profiles is feasible and may become therapeutically relevant once functional studies have been conducted.

## Methods

### Isolation, culture and characterization of FSK-MSCs

This study was approved by the Bordet Institute Ethics Committee (Belgium) and conducted in accordance with the Declaration of Helsinki (1964). Three FSK samples were obtained following a circumcision procedure and all donors and/or their parents gave written informed consent. All the procedures for the isolation, culture and characterization of FSK-MSCs were conducted according to our previously study [12]. Briefly, after the surgical procedure, FSK samples were collected into a sterile specimen container containing sterile phosphate-buffered saline (PBS; Lonza) supplemented with penicillin/streptomycin (Lonza). After a wash with sterile PBS, the FSK was sectioned longitudinally to spread the tissue and the epidermis was manually removed from the skin. The dermis was cut into small pieces and tissue dissociation was applied with enzymatic digestion by incubation for 1 h at 37 °C with 0.2 mg/mL of Liberase Research Grade solution (Roche Diagnostics). After digestion step, 10% fetal bovine serum (FBS; Sigma-Aldrich) was added to neutralize the enzymes, and the cell suspension was washed by centrifugation (800 g for 5 min at room temperature) in Dulbecco’s Modified Eagle’s Medium with low glucose (DMEM-LG; Lonza). The resulting cell pellet was then seeded in culture flasks with DMEM-LG (Lonza) supplemented with 10% FBS (Sigma-Aldrich), 2 mmol/L L-glutamine and 50 U/mL penicillin/streptomycin (both from Lonza). Cell cultures were maintained at 37 °C in a 5% CO2 humidified atmosphere. When sub-confluence (80–90%) was achieved, adherent cells were harvested by TrypLE Select (Gibco, LifeTechnologies) and expanded until the second passage (P2). The characterization of FSK-MSCs was achieved according to the ISCT criteria. Both immunophenotype and multilineage potential were thus confirmed. Briefly, the immunophenotype was determined by flow cytometry analysis using a panel of fluorochrome labelled monoclonal antibodies against membrane markers. The multilineage potential was demonstrated by culturing FSK-MSCs in appropriate induction medium and by using specific coloration to show their adipogenic, osteogenic and chondrogenic differentiation capacities (more details presented in Additional file 1).

### Fluorescence activated cell sorting analysis (FACS)

Cell sorting was conducted using a FACS-Aria (BD Biosciences, San Jose, CA) as it has been previously described [97]. The instrument quality control was checked on a daily basis throughout by using Cytometer setting and tracking beads (CS&T) and software (FACSDiva). A primary gate was placed on the scatter population and a second one for PI (Propidium iodide) (Fluka, 70,335) negative cells allowing thus to analyze only live cells excluding residual erythrocytes and debris. We also have incorporated a doublet discriminating gate based upon height versus area of the side scatter signals. These settings were then used throughout the assay. Analysis was done using the Flo-Jo program (Tree Star, Ashland, https://www.flowjo.com/).

### Detection, study and sorting of FSK-MSCs based on their ALDH activity

Fluorescent ALDH substrate (BODIPY® - aminoacetaldehyde (BAAA/ALDEFLUOR™ kit) (StemCell Technologies) is used to identify and isolate a population with high ALDH versus low ALDH enzymatic activity (hereafter referred to as ALDH^+^ and ALDH^−^) according to our previous report (see [96, 97] for further information). As a non-polar fluorescent molecule, BAAA is taken up by viable cells through passive diffusion. The BAAA is composed of 2 parts: the Bodipy molecule which contains the green fluorescence and the amino-acetaldehyde which is the substrate for ALDH1A1. BAAA could be metabolized by this isoform [98] to a carboxylate fluorescent ion BAA^−^ which is retained intracellularly, allowing thus to sort by FACS cells demonstrating high levels of ALDH activity (the assay buffer used for analysis contains an efflux inhibitor; for further information, see (http://www.aldh.org/)).

Dissociated single cells (with trypsin) were suspended in ALDEFLUOR™ assay buffer containing the BAAA (1 μM per 3 × 10^6^ cells) and incubated at + 37 °C without agitation during 50 min. ALDEFLUOR™ staining was performed three times for each sample. The sorting gate of the ALDH^+^ cells was established using DEAB-treated cells as a reference. For all subsequent procedures, samples were constantly maintained at + 4 °C to prevent efflux. ALDEFLUOR™ fluorescence was excited at 488 nm, and fluorescence emission was detected using a standard fluorescein-isothiocyanate 530/30 nm band-pass filter. All samples were thus sorted into 2 different collector tubes, namely “ALDH^+^” and “ALDH^−^”.

### Gene expression profiling by real time PCR (qPCR)

Immediately after the sorting, the cells were spin down by centrifugation. The cell lysate buffer (10 mL of BL-buffer from Promega (ref Z103c) was mixed with 10 μL of thioglycerol (ref A208B)) to perform the extraction of the total mRNA. Since the number of cells obtained after the cell sorting were roughly small for some populations, we used the RNeasy micro kit (ref 74,004) from Qiagen to ensure optimal RNA purification. We performed the reverse transcription reaction with 1 mg RNA using qScript cDNA SuperMix (Quanta Biosciences). Transcripts were quantified by qRT-PCR using 10 ng of cDNA, SYBR Green PCR Master Mix (Applied Biosystems, Lennik, Belgium) and 0.32 mM forward and reverse primers. The primers were designed with Primer Express 2.0 software (Applied Biosystems) or ProbeFinder online software (Roche) and are available in Additional file 1: Table S1. For qPCR analysis, each condition has been run in triplicate. To control variations in input RNA amounts, the GAPDH gene was used as a housekeeping gene to quantify and normalize the results. The reactions were carried out using the ABI Prism 7900 HT system (Applied Biosystems). In all cases, dissociation curves were generated and the specificity of the PCR reactions was confirmed. The comparative ΔΔCt method was used for the data analysis. To evaluate the fold change, data were normalized with the GAPDH genes to obtain the ΔCt and were after calibrated with the geometric mean of the GAPDH ΔCt to generate the ΔΔCt. Fold changes were then calculated as fold change = 2^-ΔΔCt^.

### Statistical analysis

Data are presented as mean ± standard error of the mean (SEM). Comparison between sorted fractions was evaluated with the matched paired t test. *p* values < 0.05 were considered as statistically significant. All analyses were performed with GraphPad Prism version 5.00 for windows (GraphPad Software, www.graphpad.com).

## Additional file

Additional file 1: Supplemental data. (DOC 8200 kb)

## References

[1] Forostyak S, Jendelova P, Sykova E (2013). The role of mesenchymal stromal cells in spinal cord injury, regenerative medicine and possible clinical applications. Biochimie.

[2] Zimmerlin L, Park TS, Zambidis ET, Donnenberg VS, Donnenberg AD (2013). Mesenchymal stem cell secretome and regenerative therapy after cancer. Biochimie.

[3] Dunavin N, Dias A, Li M, McGuirk J. Mesenchymal stromal cells: what is the mechanism in acute graft-versus-host disease? Biomedicine. 2017;5(3):39. 10.3390/biomedicines5030039. http://www.mdpi.com/2227-9059/5/3/39. 10.3390/biomedicines5030039 PMC5618297 28671556

[4] Dizaji Asl K, Shafaei H, Soleimani Rad J, Nozad HO (2017). Comparison of characteristics of human amniotic membrane and human adipose tissue derived mesenchymal stem cells. World J Plast Surg.

[5] Meuleman N, Tondreau T, Delforge A, Dejeneffe M, Massy M, Libertalis M (2006). Human marrow mesenchymal stem cell culture: serum-free medium allows better expansion than classical alpha-MEM medium. Eur J Haematol.

[6] Busser H, Najar M, Raicevic G, Pieters K, Velez Pombo R, Philippart P (2015). Isolation and characterization of human mesenchymal stromal cell subpopulations: comparison of bone marrow and adipose tissue. Stem Cells Dev.

[7] Rizk M, Monaghan M, Shorr R, Kekre N, Bredeson CN, Allan DS (2016). Heterogeneity in studies of mesenchymal stromal cells to treat or prevent graft-versus-host disease: a scoping review of the evidence. Biol Blood Marrow Transplant.

[8] Kang CM, Kim H, Song JS, Choi BJ, Kim SO, Jung HS (2016). Genetic comparison of Stemness of human umbilical cord and dental pulp. Stem Cells Int.

[9] Lee JM, Jung J, Lee HJ, Jeong SJ, Cho KJ, Hwang SG (2012). Comparison of immunomodulatory effects of placenta mesenchymal stem cells with bone marrow and adipose mesenchymal stem cells. Int Immunopharmacol.

[10] Hoogduijn MJ, Gorjup E, Genever PG (2006). Comparative characterization of hair follicle dermal stem cells and bone marrow mesenchymal stem cells. Stem Cells Dev.

[11] Somuncu OS, Tasli PN, Sisli HB, Somuncu S, Sahin F (2015). Characterization and differentiation of stem cells isolated from human newborn foreskin tissue. Appl Biochem Biotechnol.

[12] Najar M, Raicevic G, Andre T, Fayyad-Kazan H, Pieters K, Bron D (2016). Mesenchymal stromal cells from the foreskin: tissue isolation, cell characterization and immunobiological properties. Cytotherapy.

[13] Li Y, Zhao Y, Cheng Z, Zhan J, Sun X, Qian H (2013). Mesenchymal stem cell-like cells from children foreskin inhibit the growth of SGC-7901 gastric cancer cells. Exp Mol Pathol.

[14] Huang HI, Chen SK, Wang RYL, Shen CR, Cheng YC (2013). Human foreskin fibroblast-like stromal cells can differentiate into functional hepatocytic cells. Cell Biol Int.

[15] Veryasov VN, Savilova AM, Buyanovskaya OA, Chulkina MM, Pavlovich SV, Sukhikh GT (2014). Isolation of mesenchymal stromal cells from extraembryonic tissues and their characteristics. Bull Exp Biol Med.

[16] Chan TM, Harn HJ, Lin HP, Chou PW, Chen JY-R, Ho TJ (2014). Improved human mesenchymal stem cell isolation. Cell Transplant.

[17] Ma I, Allan AL (2011). The role of human aldehyde dehydrogenase in normal and cancer stem cells. Stem Cell Rev.

[18] Xu X, Chai S, Wang P, Zhang C, Yang Y, Yang Y (2015). Aldehyde dehydrogenases and cancer stem cells. Cancer Lett.

[19] Balber AE (2011). Concise review: aldehyde dehydrogenase bright stem and progenitor cell populations from normal tissues: characteristics, activities, and emerging uses in regenerative medicine. Stem Cells.

[20] Muramoto GG, Russell JL, Safi R, Salter AB, Himburg HA, Daher P (2010). Inhibition of aldehyde dehydrogenase expands hematopoietic stem cells with radioprotective capacity. Stem Cells.

[21] Huang EH, Hynes MJ, Zhang T, Ginestier C, Dontu G, Appelman H (2009). Aldehyde dehydrogenase 1 is a marker for normal and malignant human colonic stem cells (SC) and tracks SC overpopulation during colon tumorigenesis. Cancer Res.

[22] Tomita H, Tanaka K, Tanaka T, Hara A (2016). Aldehyde dehydrogenase 1A1 in stem cells and cancer. Oncotarget.

[23] Pors K, Moreb JS (2014). Aldehyde dehydrogenases in cancer: an opportunity for biomarker and drug development?. Drug Discov Today.

[24] Alison MR, Guppy NJ, Lim SML, Nicholson LJ (2010). Finding cancer stem cells: are aldehyde dehydrogenases fit for purpose?. J Pathol.

[25] Herzenberg LA, Parks D, Sahaf B, Perez O, Roederer M, Herzenberg LA (2002). The history and future of the fluorescence activated cell sorter and flow cytometry: a view from Stanford. Clin Chem.

[26] Friedenstein AJ, Petrakova KV, Kurolesova AI, Frolova GP (1968). Heterotopic of bone marrow. Analysis of precursor cells for osteogenic and hematopoietic tissues. Transplantation.

[27] Tondreau T, Meuleman N, Delforge A, Dejeneffe M, Leroy R, Massy M (2005). Mesenchymal stem cells derived from CD133-positive cells in mobilized peripheral blood and cord blood: proliferation, Oct4 expression, and plasticity. Stem Cells.

[28] Zuk PA, Zhu M, Mizuno H, Huang J, Futrell JW, Katz AJ (2001). Multilineage cells from human adipose tissue: implications for cell-based therapies. Tissue Eng.

[29] Campagnoli C, Roberts IA, Kumar S, Bennett PR, Bellantuono I, Fisk NM (2001). Identification of mesenchymal stem/progenitor cells in human first-trimester fetal blood, liver, and bone marrow. Blood.

[30] Hoogduijn MJ, Popp F, Verbeek R, Masoodi M, Nicolaou A, Baan C (2010). The immunomodulatory properties of mesenchymal stem cells and their use for immunotherapy. Int Immunopharmacol.

[31] Fayyad-Kazan M, Najar M, Fayyad-Kazan H, Raicevic G, Identification LL (2017). Evaluation of new Immunoregulatory genes in mesenchymal stromal cells of different origins: comparison of normal and inflammatory conditions. Med Sci Monit Basic Res.

[32] Najar M, Raicevic G, Fayyad-Kazan H, De Bruyn C, Bron D, Toungouz M (2012). Immune-related antigens, surface molecules and regulatory factors in human-derived mesenchymal stromal cells: the expression and impact of inflammatory priming. Stem Cell Rev.

[33] Vishnubalaji R, Al-Nbaheen M, Kadalmani B, Aldahmash A, Ramesh T (2012). Skin-derived multipotent stromal cells--an archrival for mesenchymal stem cells. Cell Tissue Res.

[34] Sharpe ME, Morton D, Rossi A (2012). Nonclinical safety strategies for stem cell therapies. Toxicol Appl Pharmacol.

[35] Atouf F, Provost NM, Rosenthal FM. Standards for ancillary materials used in cell- and tissue-based therapies. In: BioProcess International. 2013;11(8):12–20. http://www.bioprocessintl.com/wp-content/uploads/bpi-content/BPI_A_131108AR01_O_229785a.pdf. Accessed 1 Sept 2013.

[36] Tondreau T, Lagneaux L, Dejeneffe M, Delforge A, Massy M, Mortier C (2004). Isolation of BM mesenchymal stem cells by plastic adhesion or negative selection: phenotype, proliferation kinetics and differentiation potential. Cytotherapy.

[37] Najar M, Raicevic G, Boufker HI, Fayyad Kazan H, De Bruyn C, Meuleman N (2010). Mesenchymal stromal cells use PGE2 to modulate activation and proliferation of lymphocyte subsets: combined comparison of adipose tissue, Wharton's jelly and bone marrow sources. Cell Immunol.

[38] Kusuma GD, Abumaree MH, Perkins AV, Brennecke SP, Kalionis B (2017). Reduced aldehyde dehydrogenase expression in preeclamptic decidual mesenchymal stem/stromal cells is restored by aldehyde dehydrogenase agonists. Sci Rep.

[39] Kusuma GD, Abumaree MH, Pertile MD, Perkins AV, Brennecke SP, Kalionis B (2016). Mesenchymal stem/stromal cells derived from a reproductive tissue niche under oxidative stress have high aldehyde dehydrogenase activity. Stem Cell Rev.

[40] Sherman SE, Kuljanin M, Cooper TT, Putman DM, Lajoie GA, Hess DA (2017). High aldehyde dehydrogenase activity identifies a subset of human mesenchymal stromal cells with vascular regenerative potential. Stem Cells.

[41] Rodriguez-Torres M, Allan AL (2016). Aldehyde dehydrogenase as a marker and functional mediator of metastasis in solid tumors. Clin Exp Metastasis.

[42] Sun X, Zhu H, Dong Z, Liu X, Ma X, Han S (2017). Mitochondrial aldehyde dehydrogenase-2 deficiency compromises therapeutic effect of ALDH bright cell on peripheral ischemia. Redox Biol.

[43] De Kock J, Najar M, Bolleyn J, Al Battah F, Rodrigues RM, Buyl K (2012). Mesoderm-derived stem cells: the link between the transcriptome and their differentiation potential. Stem Cells Dev.

[44] Stanko P, Kaiserova K, Altanerova V, Altaner C (2014). Comparison of human mesenchymal stem cells derived from dental pulp, bone marrow, adipose tissue, and umbilical cord tissue by gene expression. Biomed Pap Med Fac Univ Palacky Olomouc Czech Repub.

[45] Dolatabadi S, Candia J, Akrap N, Vannas C, Tesan Tomic T, Losert W (2017). Cell cycle and cell size dependent gene expression reveals distinct subpopulations at single-cell level. Front Genet.

[46] Heo JS, Choi Y, Kim HS, Kim HO (2016). Comparison of molecular profiles of human mesenchymal stem cells derived from bone marrow, umbilical cord blood, placenta and adipose tissue. Int J Mol Med.

[47] Torensma R, Prins HJ, Schrama E, Verwiel ETP, Martens ACM, Roelofs H (2013). The impact of cell source, culture methodology, culture location, and individual donors on gene expression profiles of bone marrow-derived and adipose-derived stromal cells. Stem Cells Dev.

[48] Rasini V, Dominici M, Kluba T, Siegel G, Lusenti G, Northoff H (2013). Mesenchymal stromal/stem cells markers in the human bone marrow. Cytotherapy.

[49] Tsai CC, Hung SC (2012). Functional roles of pluripotency transcription factors in mesenchymal stem cells. Cell Cycle.

[50] Dey D, Pan G, Varma NR, Palaniyandi SS (2015). Sca-1+ cells from fetal heart with high aldehyde dehydrogenase activity exhibit enhanced gene expression for self-renewal, proliferation, and survival. Oxidative Med Cell Longev.

[51] Meng E, Mitra A, Tripathi K, Finan MA, Scalici J, McClellan S (2014). ALDH1A1 maintains ovarian cancer stem cell-like properties by altered regulation of cell cycle checkpoint and DNA repair network signaling. PLoS One.

[52] Hegab AE, Ha VL, Bisht B, Darmawan DO, Ooi AT, Zhang KX (2014). Aldehyde dehydrogenase activity enriches for proximal airway basal stem cells and promotes their proliferation. Stem Cells Dev.

[53] Muzio G, Maggiora M, Paiuzzi E, Oraldi M, Canuto RA (2012). Aldehyde dehydrogenases and cell proliferation. Free Radic Biol Med.

[54] New SEP, Alvarez-Gonzalez C, Vagaska B, Gomez SG, Bulstrode NW, Madrigal A (2015). A matter of identity - phenotype and differentiation potential of human somatic stem cells. Stem Cell Res.

[55] Najar M, Raicevic G, Jebbawi F, De Bruyn C, Meuleman N, Bron D (2012). Characterization and functionality of the CD200-CD200R system during mesenchymal stromal cell interactions with T-lymphocytes. Immunol Lett.

[56] Vishnubalaji R, Manikandan M, Al-Nbaheen M, Kadalmani B, Aldahmash A, Alajez NM (2012). In vitro differentiation of human skin-derived multipotent stromal cells into putative endothelial-like cells. BMC Dev Biol.

[57] Huang HI, Chen SK, Ling QD, Chien CC, Liu HT, Chan SH (2010). Multilineage differentiation potential of fibroblast-like stromal cells derived from human skin. Tissue Eng Part A.

[58] Espagnolle N, Balguerie A, Arnaud E, Sensebe L, Varin A (2017). CD54-mediated interaction with pro-inflammatory macrophages increases the immunosuppressive function of human mesenchymal stromal cells. Stem Cell Rep.

[59] Baer PC (2014). Adipose-derived mesenchymal stromal/stem cells: an update on their phenotype in vivo and in vitro. World J Stem Cells.

[60] Yang ZX, Han ZB, Ji YR, Wang YW, Liang L, Chi Y (2013). CD106 identifies a subpopulation of mesenchymal stem cells with unique immunomodulatory properties. PLoS One.

[61] Du W, Li X, Chi Y, Ma F, Li Z, Yang S (2016). VCAM-1+ placenta chorionic villi-derived mesenchymal stem cells display potent pro-angiogenic activity. Stem Cell Res Ther.

[62] Barcia RN, Santos JM, Filipe M, Teixeira M, Martins JP, Almeida J (2015). What makes umbilical cord tissue-derived mesenchymal stromal cells superior Immunomodulators when compared to bone marrow derived mesenchymal stromal cells?. Stem Cells Int.

[63] Pontikoglou C, Langonne A, Ba MA, Varin A, Rosset P, Charbord P (2016). CD200 expression in human cultured bone marrow mesenchymal stem cells is induced by pro-osteogenic and pro-inflammatory cues. J Cell Mol Med.

[64] Wang J, Zhu Z, Huang Y, Wang P, Luo Y, Gao Y (2014). The subtype CD200-positive, chorionic mesenchymal stem cells from the placenta promote regeneration of human hepatocytes. Biotechnol Lett.

[65] Wu CC, Liu FL, Sytwu HK, Tsai CY, Chang DM (2016). CD146+ mesenchymal stem cells display greater therapeutic potential than CD146- cells for treating collagen-induced arthritis in mice. Stem Cell Res Ther.

[66] Nauta TD, van Hinsbergh VWM, Koolwijk P (2014). Hypoxic signaling during tissue repair and regenerative medicine. Int J Mol Sci.

[67] Bartels K, Grenz A, Eltzschig HK (2013). Hypoxia and inflammation are two sides of the same coin. Proc Natl Acad Sci U S A.

[68] D'Ippolito G, Diabira S, Howard GA, Roos BA, Schiller PC (2006). Low oxygen tension inhibits osteogenic differentiation and enhances stemness of human MIAMI cells. Bone.

[69] Ohnishi S, Yasuda T, Kitamura S, Nagaya N (2007). Effect of hypoxia on gene expression of bone marrow-derived mesenchymal stem cells and mononuclear cells. Stem Cells.

[70] Kim RJ, Park JR, Roh KJ, Choi AR, Kim SR, Kim PH (2013). High aldehyde dehydrogenase activity enhances stem cell features in breast cancer cells by activating hypoxia-inducible factor-2alpha. Cancer Lett.

[71] Perin EC, Silva GV, Zheng Y, Gahremanpour A, Canales J, Patel D, et al. Randomized, double-blind pilot study of transendocardial injection of autologous aldehyde dehydrogenase-bright stem cells in patients with ischemic heart failure. Am Heart J. 2012;163(3):415–21. 421 10.1016/j.ahj.2011.11.020 22424012

[72] Perin EC, Murphy MP, March KL, Bolli R, Loughran J, Yang PC (2017). Evaluation of cell therapy on exercise performance and limb perfusion in peripheral artery disease: the CCTRN PACE trial (patients with intermittent claudication injected with ALDH bright cells). Circulation.

[73] Capoccia BJ, Robson DL, Levac KD, Maxwell DJ, Hohm SA, Neelamkavil MJ (2009). Revascularization of ischemic limbs after transplantation of human bone marrow cells with high aldehyde dehydrogenase activity. Blood.

[74] Fajardo-Orduna GR, Mayani H, Montesinos JJ (2015). Hematopoietic support capacity of mesenchymal stem cells: biology and clinical potential. Arch Med Res.

[75] Lioznov MV, Freiberger P, Kroger N, Zander AR, Fehse B (2005). Aldehyde dehydrogenase activity as a marker for the quality of hematopoietic stem cell transplants. Bone Marrow Transplant.

[76] Briquet A, Dubois S, Bekaert S, Dolhet M, Beguin Y, Gothot A (2010). Prolonged ex vivo culture of human bone marrow mesenchymal stem cells influences their supportive activity toward NOD/SCID-repopulating cells and committed progenitor cells of B lymphoid and myeloid lineages. Haematologica.

[77] Fayyad-Kazan M, Fayyad-Kazan H, Lagneaux L, Najar M (2016). The potential of mesenchymal stromal cells in immunotherapy. Immunotherapy.

[78] Abdi R, Fiorina P, Adra CN, Atkinson M, Sayegh MH (2008). Immunomodulation by mesenchymal stem cells: a potential therapeutic strategy for type 1 diabetes. Diabetes.

[79] Najar M, Raicevic G, Id Boufker H, Stamatopoulos B, De Bruyn C, Meuleman N (2010). Modulated expression of adhesion molecules and galectin-1: role during mesenchymal stromal cell immunoregulatory functions. Exp Hematol.

[80] Purandare B, Teklemariam T, Zhao L, Hantash BM (2014). Temporal HLA profiling and immunomodulatory effects of human adult bone marrow- and adipose-derived mesenchymal stem cells. Regen Med.

[81] Najar M, Raicevic G, Crompot E, Fayyad-Kazan H, Bron D, Toungouz M (2016). The immunomodulatory potential of mesenchymal stromal cells: a story of a regulatory network. J Immunother.

[82] Steimle A, Frick JS (2016). Molecular mechanisms of induction of tolerant and Tolerogenic intestinal dendritic cells in mice. J Immunol Res.

[83] Rohban R, Pieber TR (2017). Mesenchymal stem and progenitor cells in regeneration: tissue specificity and regenerative potential. Stem Cells Int.

[84] Vella JB, Thompson SD, Bucsek MJ, Song M, Huard J (2011). Murine and human myogenic cells identified by elevated aldehyde dehydrogenase activity: implications for muscle regeneration and repair. PLoS One.

[85] Lavoie JF, Biernaskie JA, Chen Y, Bagli D, Alman B, Kaplan DR (2009). Skin-derived precursors differentiate into skeletogenic cell types and contribute to bone repair. Stem Cells Dev.

[86] Wada N, Bartold PM, Gronthos S (2011). Human foreskin fibroblasts exert immunomodulatory properties by a different mechanism to bone marrow stromal/stem cells. Stem Cells Dev.

[87] Nagano M, Kimura K, Yamashita T, Ohneda K, Nozawa D, Hamada H (2010). Hypoxia responsive mesenchymal stem cells derived from human umbilical cord blood are effective for bone repair. Stem Cells Dev.

[88] Itoh H, Nishikawa S, Haraguchi T, Arikawa Y, Eto S, Hiyama M (2017). Aldehyde dehydrogenase activity helps identify a subpopulation of murine adipose-derived stem cells with enhanced adipogenic and osteogenic differentiation potential. World J Stem Cells.

[89] Itoh H, Nishikawa S, Haraguchi T, Arikawa Y, Hiyama M, Eto S (2017). Aldehyde dehydrogenase activity identifies a subpopulation of canine adipose-derived stem cells with higher differentiation potential. J Vet Med Sci.

[90] Menssen A, Haupl T, Sittinger M, Delorme B, Charbord P, Ringe J (2011). Differential gene expression profiling of human bone marrow-derived mesenchymal stem cells during adipogenic development. BMC Genomics.

[91] Lee JE, Ge K (2014). Transcriptional and epigenetic regulation of PPARgamma expression during adipogenesis. Cell Biosci.

[92] Su Y, Shen X, Chen J, Isales CM, Zhao J, Shi XM. Differentially expressed genes in PPARgamma-deficient MSCs. Mol Cell Endocrinol. 2017; 10.1016/j.mce.2017.07.037. 10.1016/j.mce.2017.07.037 PMC5792374 28774780

[93] Ronziere MC, Perrier E, Mallein-Gerin F, Freyria AM (2010). Chondrogenic potential of bone marrow- and adipose tissue-derived adult human mesenchymal stem cells. Biomed Mater Eng.

[94] Gonzalez-Fernandez ML, Perez-Castrillo S, Ordas-Fernandez P, Lopez-Gonzalez ME, Colaco B, Villar-Suarez V (2015). Study on viability and chondrogenic differentiation of cryopreserved adipose tissue-derived mesenchymal stromal cells for future use in regenerative medicine. Cryobiology.

[95] Estes BT, Wu AW, Storms RW, Guilak F (2006). Extended passaging, but not aldehyde dehydrogenase activity, increases the chondrogenic potential of human adipose-derived adult stem cells. J Cell Physiol.

[96] Dolle L, Boulter L, Leclercq IA, van Grunsven LA (2015). Next generation of ALDH substrates and their potential to study maturational lineage biology in stem and progenitor cells. Am J Physiol Gastrointest Liver Physiol.

[97] Dolle L, Best J, Empsen C, Mei J, Van Rossen E, Roelandt P (2012). Successful isolation of liver progenitor cells by aldehyde dehydrogenase activity in naive mice. Hepatology.

[98] Moreb JS, Ucar D, Han S, Amory JK, Goldstein AS, Ostmark B (2012). The enzymatic activity of human aldehyde dehydrogenases 1A2 and 2 (ALDH1A2 and ALDH2) is detected by Aldefluor, inhibited by diethylaminobenzaldehyde and has significant effects on cell proliferation and drug resistance. Chem Biol Interact.

