# Supplementary material for: Foreskin-derived mesenchymal stromal cells with aldehyde dehydrogenase activity: isolation and gene profiling
Source: BMC Cell Biol. 2018 Apr 6;19:4. doi: 10.1186/s12860-018-0157-0 (PMC5889569; doi:10.1186/s12860-018-0157-0)
Supplement: Supplementary file 1 — Supplemental data. (DOC 8200 kb) [file 12860_2018_157_MOESM1_ESM.doc]

Foreskin derived mesenchymal stromal cells with aldehyde dehydrogenase activity: isolation and gene profiling

**Additional file 1**

***Osteogenic differentiation:*** 5,000 cells/well were plated in a 24 well plate with culture medium. After 5 days, medium was completely discarded and replaced by osteogenic medium (StemMACS OsteoDiff Media, Miltenyi Biotec). Cells were fed weekly with complete replacement of osteogenic medium. After 21 days, the mineralization of the extracellular matrix was assessed by Alizarin Red staining. Cells were washed in phosphate-buffered saline (PBS) and fixed in 70% ethanol at room temperature for 5 min followed by several washes in H2O. Cells were stained in 40mM Alizarin red (Sigma-Aldrich) pH= 4.2 for 15 min at room temperature, rinsed in H2O, and then air-dried. The red staining was examined by light microscopy.

***Adipogenic differentiation:*** 5,000 cells/well were plated in a 24well plate with culture medium. After 5 days, medium was completely discarded and replaced with adipogenic medium (StemMACS AdipoDiff Media, Miltenyi Biotec). Cells were fed weekly with complete replacement of adipogenic medium. At day 7, cells were stained with Oil Red O solution (Sigma) after fixing (8% formaldehyde). Lipid vacuoles were then observed by light microscopy.

***Chondrogenic differentiation:*** 150,000 cells were cultured in the tip of a 15 ml conical tube (Greiner) to enable cell culture in micromass with chondrogenic medium (StemMACS ChondroDiff Media, Miltenyi Biotec). Cells were resuspended carefully and cultured at 37°C, 5% CO2, humidified atmosphere with cap slightly screwed. Half of the chondrogenic medium was replaced weekly. At day 21, aggregates were stained with Alcian blue (Sigma) to highlight cartilage proteoglycans. In some cases, cryosectioned pellets were stained with Alcian blue to confirm chondrogenic differentiation.

**Figure S1**

**Table S1: qRT-PCR primers**

| **Transcripts** | **Forward** | **Reverse** |
| --- | --- | --- |
| **Cell Cycle** |  |  |
| p53 | AGGCCTTGGAACTCAAGGAT | CCCTTTTTGGACTTCAGGTG |
| p21 | CGAAGTCAGTTCCTTGTGGAG | CATGGGTTCTGACGGACAT |
| p16 | TGCCTTTTCACTGTGTTGGA | TGCTTGTCATGAAGTCGACAG |
| pRB | TCCTGAGGAGGACCCAGAG | AGGTTCTTCTGTTTCTTCAAACTCA |
| CDC25A | CGTCATGAGAACTACAAACCTTGA | TCTGGTCTCTTCAACACTGACC |
| FosB | CCGAGAGGAGACGCTCAC | CTGCTGCTAGTTTATTTCGTTCC |
| STAT1 | GACTGAGTTGATTTCTGTGTCTGAA | ACACCTCGTCAAACTCCTCAG |
| CCNA | GGTACTGGAGTCCGGGAACC | GAAGATCCTTAAGGGGTGCAA |
| CCNB | CCTCCGGTGTTCTGCTTC | TTCAGCATTAATTTTCGAGTTCC |
| CCNE | CTTCACAGGGAGACCTTTTAC | CATTCAGCCAGGACACAATAG |
| CDK1 | TGGATCTGAAGAAATACTTGGATTCTA | CAATCCCCTGTAGGATTTGG |
| CDK2 | GCTAGCAGACTTTGGACTAGCCAG | AGCTCGGTACCACAGGGTCA |
| **Hypoxia** |  |  |
| HIF1α | TGGAATGGAGCAAAAGACAA | CAGCTGTGGTAATCCACTTTCA |
| HIF2α | CATCATGCGACTGGCAAT | GCTTCGGACTCGTTTCAGA |
| GLUT1 | CTTCCTACCCAACCACTCAAA | CCCTCTCCTCCCTGCACT |
| **Stemness** |  |  |
| SOX2 | CCATCCACACTCACGCAAAA | CCCCCAAAAAGAAGTCCCAA |
| REX1 | CCCTGGACTGCGAGATGT | AGGCTTCACGAAGGTGTCAT |
| NANOG | ACAACTGGCCGAAGAATAGCA | GGTTCCCAGTCGGGTTCAC |
| OCT4 | CTTCGCAAGCCCTCATTTCAC | TTGATGTCCTGGGACTCCTCC |
| **Angiogenesis** |  |  |
| ANG1 | TTCCTGTCAAGTCATCTTGTGAA | TTTTATTAAGGTTGCACATCCAAG |
| ANG2 | GGCAGCGTTGATTTTCAGAG | TTGCGAAACAAACTCATTTCC |
| FLT1 | ACATTGGCCACCATCTGAAC | GCAGTATTCAACAATCACCATCA |
| VEGF | CTACCTCCACCATGCCAAGT | GCAGTAGCTGCGCTGATAGA |
| **Phenotype** |  |  |
| CD54 | AGTGATCAGGGTCCTGCAA | GGGAGGGAGTCCTCCAATAC |
| CD58 | CCAATGCATGATACCAGAGCAT | CCAATGCATGATACCAGAGCAT |
| CD106 | GGCTGTGAATCCCCATCTT | AATTGGTCCCCTCACTCCTC |
| CD146 | GGGTACCCCATTCCTCAAGT | CAGTCTGGGACGACTGAATG |
| CD200 | TCTACCTACAGCCTGGTTTGG | TGGGTCACCACTTGCACTT |
| **Immuno** |  |  |
| GAL1 | AAGCTGCCAGATGGATACGAA | CGTCAGCTGCCATGTAGTTGA |
| COX1 | CCTGCAGCTGAAATTTGACCCA | ACCTTGAAGGAGTCAGGCATG |
| COX2 | GCTCAAACATGATGTTTGCATTC | GCTGGCCCTCGCTTATGA |
| HGF | CAATGCCCTCTGGTTCCCCTT | AGGCAAAAAGCTGTGTTCGTG |
| LIF | TGAAAACTGCCGGCATCTGA | CTGTGTACTGCCGCCAAGA |
| **Hematopoiesis** |  |  |
| IL-6 | AAATTCGGTACATCCTCGACGG | GGAAGGTTCAGGTTGTTTTCTGC |
| IL-8 | CTGTTAAATCTGGCAACCCTAGTCT | CAAGGCACAGTGGAACAAGGA |
| SCF | AGCCAGCTCCCTTAGGAATG | CGAGTGGGTCTAGCGGAAAG |
| MMP2 | TGATCTTGACCAGAATACCATCGA | GGCTTGCGAGGGAAGAAGTT |
| SDF1 | CTGTGCCCTTCAGATTGTAGCC | CTGTAAGGGTTCCTCAGGCG |
| **Osteogenesis** |  |  |
| OSX | TAGGCAGCAGCAGTAGCAGA | TCTGACTCCAGAGTCCTTGCT |
| BSP | TACACGGGCGTCAATGAATA | AGGTTCCCCGTTCTCACTTT |
| RUNX2 | TTACTTACACCCCGCCAGTC | TATGGAGTGCTGCTGGTCTG |
| OPN | TTGCAGTGATTTGCTTTTGC | GCCACAGCATCTGGGTATTT |
| OPG | GGCAACACAGCTCACAAGAA | CGCTGTTTTCACAGAGGTCA |
| **Adipogenesis** |  |  |
| AdipoQ | GCTCTGTGCTCCTGCATCTG | GAGTCCATTACGCTCTCCTCC |
| PPARγ | CACAAGAACAGATCCAGTGGTTGCAG | AATAATAAGGTGGAGATGCAGGCTCC |
| KLF2 | CATCTGAAGGCGCATCTG | CGTGTGCTTTCGGTAGTGG |
| KLF5 | GGCTTTACTCAAGCAGATCTCATC | CCCTACCCATGTTGAGACG |
| CEBPδ | TGACAGCCTCGCTTGGACG | CTCTCGTCGTCGTACATGGC |
| CEBPα | TATAGGCTGGGCTTCCCCTT | AGCTTTCTGGTGTGACTCGG |
| **Chondrogenesis** |  |  |
| COL2α1 | CCCATCTGCCCAACTGACC | CCAGTCCGTCCTCTTTCACC |
| ACAN | GTGAGGAGGACATCACCGTC | AAGGCAGTGGCCCCTATTTC |
| SOX9 | GTACCCGACACTTGCACAAC | TCGCTCTCGTTCAGAAGTCTC |
| COMP | CAGGGAGATCACGTTCCTGA | GGCCGGTGCGTACTGAC |
| **Housekeeping** |  |  |
| GAPDH | AATCCCATCACCATCTTCCA | TGGACTCCACGACGTACTCA |
